# Do remittances promote financial development in Africa?

**DOI:** 10.1186/s40064-016-2658-7

**Published:** 2016-07-07

**Authors:** Nana Kwasi Karikari, Sam Mensah, Simon K. Harvey

**Affiliations:** University of Ghana Business School, Accra, Ghana

## Abstract

The paper seeks to establish whether or not remittances promoted financial developments and explore the traceable causality between remittances and financial developments in some countries in Africa. We examine the association between remittances received and how they affect the availability of credit to private sector, bank deposits intermediated by financial institutions and money supply. We also question whether the development in the financial sector causes higher levels or otherwise of remittances received. This paper uses data on remittance flows to 50 developing countries in Africa from 1990 to 2011 to explore the nexus. The study uses fixed effects and random effect estimations as well as Vector Error Correction Model method on the panel data. The study shows that remittances promote certain aspects of financial development to some extent and better financial system foster receipts of remittances. The effect of causality is seen in the short run and not in the long-run. The study alludes to literature that remittances could promote financial development in the short run and the development of the financial sector helps increase the propensity to remit via formal channels.

## Background

Efficient financial systems influence the rate of savings, leading to improved investment decisions and eventually to higher long-run growth rates (Schumpeter 1912; McKinnon 1973). The positive effect of financial development on growth has been extensively documented (Rajan and Zingale 1998; Levine 1997, 2004; Levine et al. 2000; Beck et al. 2000). Through the availability of credit at a lower cost and improved saving propensities, investments are very likely to improve which foster economic growth. In developing countries, specifically in Africa, the cost of funding can be deemed as very high. Individual, households and entrepreneurs are known to resort to other sources rather than funding via formal means from financial institutions. Monies remitted by relatives who reside overseas with better living conditions form a major part of these alternative sources of funding for the private sector.

In recent times, monies remitted to most developing countries worldwide can be seen to have grown intensely from US $68.5 billion in 1990 to US $440.0 billion in 2010 (World Bank 2011). A substantial amount of research undertaken has indicated that remittances have become the second largest source of external finance for developing countries after foreign direct investment (FDI) and about double the amount of official aid received, both in absolute terms and as a percentage of GDP (Aggarwal et al. 2010; Yang 2011). Evidence shows that in 2010, worldwide remittance flows of US $325 billion were to developing countries, an amount that constituted more than 10 percent of gross domestic product (GDP) in many developing countries (Nyamongo et al. 2012). With all the growth in the levels of remittances, both in absolute dollars and percentages, the questions we ask are, whether and how do these funds help improve the availability of credit to recipients? Does it increase their saving propensities or enable them have access to and improve the formal financial infrastructure in the recipient countries?

A number of studies have analyzed the developmental impact of remittances along various dimensions. The extent of the impact of remittances include poverty reduction, narrowing of the inequality gap, education, infant mortality, entrepreneurship and finally growth (Giuliano and Ruiz-Arranz 2009; Vaaler 2011). The concentration of this study is the ability of the recipients of these remittances to channel these monies for financial inclusion, via deposits and availability of credit which may result from deferred consumption of received remittances. According to Aggarwal et al. (2010), remittances are usually substantial in amounts and that recipients might have a need for financial products that allow them to save some of these funds for later consumption as well as gain some amount of interest earnings from the savings and boost the financial sector.

The theory, which we allude to, posits that through the channelling of remittances via formal means, banks will be able to extend banking services or products and proceed further with other investment opportunities to recipients who may be unaccustomed with banking and create adequate financial intermediation. This will be of great value to the recipients of the remitted funds by creating any further wealth. The availability of credit to the private sector can be theorised by the increase in interaction of individuals with financial institutions. This interaction can be as a result of receipts of migrant funds, for which the excess of funds over consumption increases the propensity to save, thus allowing recipient individuals, the opportunity to be introduced to financial products and services, thus deepening financial sector development.

From a contrasting perspective, Calderon et al. (2007), indicates remittances can reduce credit demands and “have dampening effect on the credit markets.” Again, “a rise in remittances might not translate itself into an increase in credit to the private sector if the flows from the remittances are channelled to finance the government or if the banks are reluctant to lend and prefer to hold liquid assets.” Chami et al. (2003) also postulate that remittances make recipients who receive remittances through informal channels, sidestep the many financial requirements in acquiring capital, which are seen as constraints (Giuliano and Ruiz-Arranz 2009). This makes less use of the financial sector services and products which in turn do not promote the financial sector.

Despite the above contrasting view, very recent studies (Vaaler 2011, 2013) explained that the entrepreneurial potentials for migrant entrepreneurs are better poised to succeed. This is due to their ability to transfer capital from the host countries to their home countries in the form of remittances and gaining financial advice from institutions. Aggregate level impact of remittances can then be said to be inconclusive. We endeavour to look at the African perspective on the impacts of remittances, not from the micro-household level, but an aggregate impact on various aspects of financial development in Africa.

Most African countries are arguably left out of the equation on the impact on financial development of remittances received. Asia and the LACs have been known to receive higher amount of remittances. Though Aggarwal et al. (2010), conducted a good study on remittances and financial development on 109 countries, continental variations and effect of remittances may result in generalized conclusions which could have been distinct for the African continent.

Due to the less developed nature of the African continent, there are staggering records of migration to more developed areas. Data from World Development Indicators (World Bank) and the International Monetary Fund (IMF) database indicate that the average remittances to African countries have grown over US $250 million in 1990 to over US $1.4 billion in the year 2011. However, a fall was seen in the year 2008 and 2009 which could be explained by the years’ financial downturn in most developed countries, which are the source of most remittances. For this reason, this study finds it important to include the recent recession period. A fall in remittances could have impacted on the level of improvement or otherwise in the financial sector of recipient countries. Remittances, despite the attention, are deemed to be severely understated since most of the migrants’ funds sent home are done mainly through informal channels as these channels do not require any formal documentation and sometimes void of transaction costs (Gibson et al. 2005; Freund and Spatafora 2008).

Previous research had indicated the use of credit to private sector and bank deposits, separately, as percentages of GDP, as measures of financial development. This study recognises that these measures may be limited to accessibility to formal financial services. This study then goes further to include quasi money (M2) or money supply as a measure of financial development, in addition to the previously known measures to have a better view of remittance effects on various proxies of financial development. This stems from the argument that the financial structure of African countries is considered under developed. The inclusion of money supply serves a greater purpose, as it creates a wider bracket for financial inclusion as compared to only having credit to private sector and bank deposits as measures of financial development. Again we include the quasi money (M2) to find out if it may behave differently from the other proxies of financial development. From the foregoing, our study then hypothesizes that, migrants remittances are thus very likely to promote the availability of credit financing to the private sector, increase deposits and money supply severally as measures of financial development in Africa.

With the presence of greater financial intermediation and financial sector reforms, all amounting to improved financial sector development, we hypothesize that financial development has the propensity to encourage the remittances of funds from developed countries to developing countries and even amongst developing countries. With improved financial sector growth, increased competition will reduce transaction cost. We hypothesize that financial inclusion could be widened through the attraction of such funds as remittances into the financial sector, rather than for mere consumption which are short lived if not used for entrepreneurial purposes. This research then again adds to existing literature to find out if there is any reverse causality traceable among financial development and remittance inflows to Africa.

This study contributes to academic research, by extending empirical studies on the links that exist between funds remitted by migrants abroad to relatives at home in Africa. It shows the availability and exposure to financial intermediary services in terms of credits to the private sector and propensity to create more financial wealth which, for the purposes of this study, focuses on deposits of remitted funds. The study also concentrates on the level of quasi money, in addition with credit to private sector and bank deposits, all as percentages of GDP, as indications or proxies of financial sector development as put forward by some studies (Demirguc-Kunt et al. 2011; Gupta et al. 2009; Aggarwal et al. 2010). To practice and policy, there seems to be a recent surge in the adoption of money transfer systems by various financial institutions. This study is necessary for a better understanding of the benefits of remittances and provides an empirical support for the efforts by financial institutions and policy makers to encourage the remitting of migrants’ funds through formal channels

The rest of the paper is organized as follows: Section two is devoted to literature review, the model is presented in section three, empirical evidence is presented in section four and finally, concluding remarks in section five.

## Literature review

This research seeks to look at the aggregate impact of remittances on the financial development of selected emerging and developing economies in Africa. How and whether or not remittances might affect financial development, particularly in the availability of credit, saving and thus investment nature as a creation of wealth and the circulation of money in Africa. This is unclear in past literatures (Gupta et al. 2009). Studies have been carried out and various reasons have been given for migrants remittances. Yang (2011) put forward, as Stark (1995) that remittances are based on altruistic motives as well as for insurance purposes against unforeseen circumstances (Gubert 2002). Clarke and Wallsten (2004), using panel data found that remittances from migrants overseas were to the tune of over twenty percent (20 %) for relief purposes of damages caused by disasters in Jamaica in 1992. Ilahi and Jafarey (1999) put forward that some of the reasons for remittances are to settle financial debts for migrants education and the cost of the migrants journey to developed countries. Apart from the survival reasons that remittances are purported to have for recipients, the funds received are, according to literature, meant for investment purposes. Remittances may be intended to fund future investments in their home countries or to pay for monitoring or administration of investment assets such as small businesses or the purchase of a land for housing to improve the living conditions of recipients. Investments in human capital in the form of education or physical capital (Yang 2008) are some reasons for the increase in these remittances over the years. Creating wealth, migrants’ funds sent back home may be intended for setting up investments or businesses (Vaaler 2011).

A number of studies have been carried out concerning the diverse impacts of migrants transferred funds. In geographical contexts, researches carried out in most developing countries in the Asia, Latin American and the Caribbeans have concentrated on the impact of remittances on economic growth as a whole and on specific issues such as poverty, micro levels of investments and entrepreneurship (Ratha 2003; Levine 2004; Spatafora 2005; Adams and Page 2005; Mundaca 2009; Noman and Uddin 2011). World Bank (2006) indicated that there are certainly economic benefits to the recipient countries that can be derived from remittances, and in total account for a significant portion of economic growth. Other research has indicated that migrants through remitted funds have been found to contribute to raising living standards of those left behind (Adams and Page 2005; Acosta et al. 2007) as well as increase return to human capital investments (Mountford 1997; Stark and Wang 2002).

Other studies (Faini 2007; Barajas et al. 2010) have both theoretically and empirically shown the positive effects that remittances have on the economy in aggregate levels. Vaaler (2011) investigated the relationship between remittances and the capital availability, creation of a new business and economic internationalization in 61 developing countries for 2002–2007 period. He alludes to the fact that there were positive effects of remittances on venture-funding. According to de Haas (2006), part of remittances that are received by individual recipients or households may be used for savings or investments. Giuliano and Ruiz-Arranz (2009) showed that in some countries with underdeveloped financial systems remittances are used to overcome credit and liquidity constraints and are invested into small business development. As postulated by Nyamongo and Misati (2011) as well as Aggarwal et al. (2010), remittances that are sent through formal channels, avenues or procedures greatly impact immensely on growth of financial sector and the economy.

The above not withstanding, extent of literature on motives and the varied impacts of remittances, cannot be said to be exhaustive. This paper extends the literature on how these remittances on aggregate level, influence the financial sector development in terms of credit availability to the private sector, levels of deposits and finally, the level of quasi money in some developing countries in Africa.

### Bi-directional causality between remittances and financial development

In the study of remittances at both the micro and macro levels, the concept of reverse causality has always been an issue. Yang (2011) at the micro level indicated that investments funded by remittances could raise household income, leading to a positive relationship, or remittances may reduce recipients need for other sources of income, resulting in a negative relationship. At the macro level, Motelle (2008), tested for the existence of reverse causalities between remittances and economic growth, with the argument that improved economies created a viable environment for business, for which remitted funds were a form of start-up capital. Calderon and Liu (2003) sought the presence of a bi-directional relationship between financial development and economic growth. The nexus between remittances and financial development in previous research has indicated the tendency for a bi-directional causality. According to Aggarwal et al. (2010), despite the cause of financial sector development by remittances, larger records of remittances may be accounted for due to the fact that, higher financial development leads to higher measurements of funds remitted through formal channels. On the reverse, improved financial sector development could create competition which will in turn reduce the cost of transmitting funds, resulting in increased remittances. This study endeavoured to find out a bi-directional causality between remittances and financial sector development and therefore posed the queries: “Do remittances promote financial development? Or does financial development propel the higher inflows of remittances?”

## Methods

### Hypotheses, data, sample and sources

The study primarily investigates whether migrants’ remittances received in Africa promote financial development. This study adopts three measures or proxies for financial development, the purposes of covering a wider range of impact. These proxies are credit to private sector, bank deposits, and money supply, all and severally as a percentage of gross domestic product (GDP). We first of all hypothesize that remittances promote the availability of credit, deposits and money supply. We again, based on the argument of reverse causality, hypothesize that the various proxies for financial sector development help attract more remittances through formal channels. To test these hypotheses, we analyse data gathered from 50 developing countries in Africa, observed from 1990 to 2011. We begin our observation from 1990 due to the increased levels of migration from Africa to the Americas and Europe and in the 2000’s because of increased levels of remittances over other assistance as well as the records of remittances via formal channels. We use 1990–2011 in order to obtain estimates of the impact of remittances over the last decade to account for the fact that recent remittances data are likely to be more accurate relative to statistics from the beginning of the sample, when less attention was given to these types of flows. All data that were used for this were attained from the World Development Indicators (WDI) and the International Monetary Fund (IMF) databases. Table 1 shows the list of the variables used, their respective categories, their descriptions and measurements.

**Table 1:** Definition of variables

Category	Variable name and simple	Variable description and measurement
Dependent variables	Credit to private sector to GDP (CRD)	The ratio of bank credit to the private sector expressed as a percentage of GDP
	Bank deposit to GDP (BDP)	Share of bank deposits expressed as a percentage of GDP
	Money supply (M2) to GDP	Measure of money supply, quasi money, M2, which is M1 plus savings and small time deposit
Key independent variable	Remittances (Rem_it_)	The level of remittances of country i at time t. measured as the ratio of official remittances received as a percentage of gross domestic product
Control variables	Gross Domestic Prod. (LogofGdp_it_)	The natural log of gross domestic product in constant US dollars. The size of the economy is captured by the log of GDP inconstant United States dollars
	Per Capita GDP (PCGdp_it_)	Per capita gross domestic product of country *i* at time *t*. Per capita GDP is the gross domestic product divided by the population of each country, measured in constant dollars
	Inflation (INF_it_)	Level of inflation of country *i* at time *t* measured as the change in the gross domestic product deflator
	Exports (Exp_it_)	Total export of country *i* at time *t*. The measure level of current account openness
	Foreign Direct Investment (FDI_it_)	Level of foreign direct investment of country *i* at time *t*. This measures for current and capital account openness in the recipient country

This table presents variable names, descriptions and measurements

### Model specification

To empirically find out if remittances do promote financial developments of countries in the African continent, considering regional differences for the period from 1990 to 2011, this study then uses the panel analytical methods to achieve the set objectives. The estimation model is thus:

1 $$FD_{it} = \alpha_{i} + \beta_{1} \text{Re}\;m_{it} + \beta_{2} Loggdp_{it} + \beta_{3} Pcgdp_{it} + \beta_{4} Inf_{it} + \beta_{5} Fdi_{it} + \beta_{6} Exp_{it} + \varepsilon_{it}$$

In Eq. (1) above, financial development (FD) is measure with three proxies as earlier stated. *FD* in country *i* of the year *t* is regressed on an intercept ($\alpha$), remittances ($\text{Re} m$) and other control variables known to affect the level of financial development sector in developing countries.

There exists a simultaneity effect that results from the independent variable, remittances, being endogenous to the model. In Gupta et al. (2009), the use of instrumental variables, such as source country variables, to solve the issues of endogeneity did not yield the needed results. This could be attributed to the sample of that study. This study however uses a broader sample from Africa with a different method to address the issues of endogeneity. The paper then, considering other estimation techniques, uses a structural Panel Vector Auto regression (PVAR) model which combines the traditional vector auto regression with a panel data approach while allowing for some variables to be held constant as control variables. This paper seeks to address the potential endogeneity that is deemed to arise since remittances; an independent variable may be correlated with the dependent variable with much concentration on the existence of reverse causality (Stock and Watson 2001).

2 $$FD_{it} = \alpha_{i} + \sum\limits_{j = 1}^{k} {\beta_{1j} FD_{it - j} } + \sum\limits_{j = 0}^{k} {\beta_{2j} \text{Re}\;m_{it - j} } + \sum\limits_{j = 1}^{5} {\beta_{3j} X_{itj} } + \varepsilon_{it}$$

3 $$\text{Re}\;m_{it} = \alpha_{i} + \sum\limits_{j = 1}^{k} {\lambda_{1j} FD_{it - j} } + \sum\limits_{j = 0}^{k} {\lambda_{2j} } \text{Re}\;m_{it - j} + \sum\limits_{j = 1}^{5} {\lambda_{3j} X_{itj} } + \varepsilon_{it}$$

We break financial development into the various proxies, which are credit to private sector (CRD), bank deposits (BDP) and money supply (M2).

#### Model 1

a $$CRD_{it} = \alpha_{i} + \sum\limits_{j = 1}^{k} {\beta_{1j} CRD_{it - j} } + \sum\limits_{j = 0}^{k} {\beta_{2j} \text{Re}\;m_{it - j} } + \sum\limits_{j = 1}^{5} {\beta_{3j} X_{itj} } + \varepsilon_{it}$$

b $$\text{Re}\;m_{it} = \alpha_{i} + \sum\limits_{j = 1}^{k} {\lambda_{1j} CRD_{it - j} } + \sum\limits_{j = 0}^{k} {\lambda_{2j} } \text{Re}\;m_{it - j} + \sum\limits_{j = 1}^{5} {\lambda_{3j} X_{itj} } + \varepsilon_{it}$$

#### Model 2

c $$BDP_{it} = \alpha_{i} + \sum\limits_{j = 1}^{k} {\beta_{1j} BDP_{it - j} } + \sum\limits_{j = 0}^{k} {\beta_{2j} \text{Re}\;m_{it - j} } + \sum\limits_{j = 1}^{5} {\beta_{3j} X_{itj} } + \varepsilon_{it}$$

d $$\text{Re}\;m_{it} = \alpha_{i} + \sum\limits_{j = 1}^{k} {\lambda_{1j} BDP_{it - j} } + \sum\limits_{j = 0}^{k} {\lambda_{2j} } \text{Re}\;m_{it - j} + \sum\limits_{j = 1}^{5} {\lambda_{3j} X_{itj} } + \varepsilon_{it}$$

#### Model 3

e $$M2_{it} = \alpha_{i} + \sum\limits_{j = 1}^{k} {\beta_{1j} M2_{it - j} } + \sum\limits_{j = 0}^{k} {\beta_{2j} \text{Re}\;m_{it - j} } + \sum\limits_{j = 1}^{5} {\beta_{3j} X_{itj} } + \varepsilon_{it}$$

f $$\text{Re}\;m_{it} = \alpha_{i} + \sum\limits_{j = 1}^{k} {\lambda_{1j} M2_{it - j} } + \sum\limits_{j = 0}^{k} {\lambda_{2j} } \text{Re}\;m_{it - j} + \sum\limits_{j = 1}^{5} {\lambda_{3j} X_{itj} } + \varepsilon_{it}$$

### Estimation strategy

For purposes of this research, countries that have their share of remittances to GDP to be equal to 1.0 % or higher are considered for inclusion. Estimations are undertaken with the use of Stata Release 13 software for the preliminary estimations and Eviews for latter estimations. We conduct and initial fixed effects and random effects estimations to establish the effect of remittances on the various proxies of financial development. We then use the panel vector autoregressive estimations to assess the bi-directional causality and address issues of endogeneity present with initial estimations. Another method of estimation that can be used for panel data analysis is the system generalized method of moments of Blundell and Blond (1998) for dynamic panel data. The system GMM has been proven to be more efficient with short time series. The system GMM considers the differencing of the variables using a few lags. One of the limits of this estimator is the asymptotic weakness of its precision and that of the instruments which involve considerable bias in finite samples.

This study uses a different methodology from previous studies on the linkages between remittances and financial development. As mentioned above, we test for the existence of a long run relationship using the Johansen panel cointegration test and subsequently use the Vector Error Correction Model (VECM). The study mainly uses the Akaike Information Criteria (AIC) to find the appropriate number of lags effective for the study.

## Results

Remittances expressed as a share of GDP have been seen to be very heterogeneous across the African continent. The World Bank in 2013 reported that the average share of remittances to GDP in Africa equalled 3.0 %. Below in Table 2, are some top recipient African countries in terms of share of remittances to GDP averaged for the 2005–2011 period and the descriptive statistics of the variables.

**Table 2:** Average shares of remittances to GDP (Top 15 African countries) *Source* Compiled from research data

Countries	Average (2005–2011) (%)
Lesotho	35.4
Nigeria	10.4
Senegal	10.4
Cape Verde	10.4
Togo	10.3
The Gambia	9.0
Liberia	7.9
Morocco	7.7
Egypt	5.3
Guinea Bissau	5.1
Tunisia	4.4
Mali	4.4
Uganda	4.3
Benin	3.6
Sudan	3.6

The descriptive statistics in Table 3 above clearly shows the nature of the data that was employed for the study. The various proxies of financial development show high percentages to GDP. The highest being money supply, clearly indicates the prevalence of that variable in the African continent compared to the other proxies of financial development. The average of remittances as a percentage of gross domestic product (GDP) from the sample is 4.0119, which is to be considered high. The details of the other variables are all seen to be in range. However, the Shapiro–Wilk test for normality rejects the null hypothesis of normality and suggests that all the variables are not normally distributed. The table exhibits that the various variables have varying numbers of observations due to the unbalanced nature of the panel data that are used in the estimations. Granted that the data spans across fifty (50) countries and over 20 years, missing data points could be said to be inevitable.

**Table 3:** Descriptive statistics

Variable	Obs.	Mean	Median	Std. Dev.	Min	Max	Shapiro–Wilk
CRD	996	27.1761	19.5365	22.2356	−0.0019	201.5771	11.43***
BDP	997	23.5619	16.9534	19.1251	0.7152	144.6355	11.42***
M2	1022	32.9913	24.3781	23.6300	0.8306	151.5489	11.82***
REM	821	4.0119	1.6503	8.3421	0.0002	78.5704	13.96***
LOGGDP	1071	9.6751	9.6611	0.6779	8.0994	11.6040	4.352***
PCGDP	1071	1346.213	513.3625	2204.502	64.8101	23,511.28	14.24***
INF	1046	30.6025	7.3539	234.2625	−10.0088	5399.526	15.89***
FDI	1042	3.4696	1.7108	5.7311	−6.8976	60.2826	13.76***
EXP	1057	29.8862	27.1386	16.3537	1.9457	91.5139	8.724***

It is seen that none of the variables but the three measures used as proxies for financial development, namely, credit to private sector to GDP (CRD), bank deposits to GDP (BDP) and quasi money to GDP (M2) exhibit multicollinearity among one another, as represented in Table 4 below. However, these proxies are never used together in a single regression model which could have created biases in the results due to multicollinearity. The correlation between these variables being strongly positive but less than 1 suggests that these variables are different in themselves but the behavior of pattern between them is to a greater extent similar or that these variables are generated from the other. Bank credit to the private sector as a percentage of GDP could be said to be derived from bank deposits which are both measures of financial development. The correlations between quasi money and the other measures of financial development used in the study is deemed high. This is arguably as a result of excess funds in circulation finding their way into the formal banking sector.

The research assessed the stationary nature of the unbalanced panel data, the research adopted the Augmented Dicker Fuller unit root test as well as the Philips Peron unit root test. As represented in Table 14 in the Appendix, some of the variables are stationary with the raw data, whereas others in Table 14 were stationary at 1st differencing. The variables that were differenced were prefixed with (D).

**Table 4:** Correlation matrix: test of multicollinearity

	CRD	BDP	M2	REM	LOGGDP	PCGDP	INF	FDI	EXP
CRD	1								
BDP	0.772**	1							
M2	0.676**	0.822**	1						
REM	0.021	0.125**	0.137**	1					
LOGGDP	0.375**	0.366**	0.355**	−0.140**	1				
PCGDP	0.152**	0.243**	0.226**	−0.105**	0.377**	1			
INF	−0.025	−0.070*	−0.044	−0.024	−0.012	−0.036	1		
FDI	−0.071*	0.023	0.058	0.260**	−0.069*	0.049	0.039	1	
EXP	0.105**	0.170**	0.083**	0.060	0.140**	0.477**	0.004	0.226**	1

 Tables 5 and 6 below report the results from both the random and the fixed effects panel regressions. This paper conducts estimations including these country and time fixed effects as well as random effects which accounts for unobserved country characteristics for common and uncommon shocks and events across the countries under study. These estimations solve the problems that may arise as a result of omitted factors that can explain both the advancement of remittances and of financial development and could have led to biases in estimating the impact of remittances on financial development. Table 5 reports on estimations on all countries used in the study. Estimations of countries with remittances as a percentage to GDP, greater than the fiftieth percentile of the data gathered, is represented in Table 6.

**Table 5:** Results from fixed and random effects estimation—all countries panel estimations—[All recipient countries]

Dependent variables	Fixed effects			Random effects		
	CRD	(D)BDP	(D)M2	CRD	(D)BDP	(D)M2
*Independent variables*						
(D) REM	−0.0617*	0.1131	0.0686**	−0.3203*	0.0989	0.1150**
	[−1.95]	[1.07]	[0.08]	[−1.96]	[1.04]	[1.98]
(D) LOGGDP	−9.1839	−11.944***	−12.6366***	−16.9781	−11.8468***	−3.5986*
	[−2.50]	[‘−3.57]	[−4.11]	[−1.52]	[−3.63]	[−1.77]
(D) PCGDP	0.0061**	0.0015**	0.0012***	0.0062	0.0015***	0.0012***
	[0.69]	[2.05]	[0.12]	[0.24]	[1.98]	[1.98]
INF	−0.0694*	−0.0746***	0.0765***	−0.0677*	−0.0662**	−0.0389***
	[−0.13]	[−6.11]	[−7.22]	[−1.75]	[−5.94]	[−6.77]
FDI	−0.3341***	0.0257	0.0195*	−0.3051***	0.03504	0.0322**
	[−3.38]	[0.85]	[0.71]	[−3.12]	[1.33]	[2.40]
EXPORTS	−0.0477	0.018558	0.0625**	−0.0606	0.0047	0.0019
	[−0.57]	[0.73]	[2.71]	[−0.80]	[0.25]	[0.30]
Constant	35.0054***	0.97447	−0.1071	37.6569***	1.4235*	0.8636***
	[13.28]	[1.22]	[−0.15]	[6.45]	[1.84]	[3.59]
R-squared	0.2142	0.4618	0.4935	0.2513	0.4704	0.5025
Number of observations	716	691	718	716	691	718
Number of countries	48	48	48	48	48	48
F-statistic	12.01	9.89	15.09			
Prob (F-statistic)	0.0001	0.0000	0.0000			
Wald chi 2				27.56	18.13	12.77
Prob > chi 2				0.0001	0.0000	0.0000

Absolute values of t-statistics are in brackets []. The symbol *, **, and *** denote significance at the 10, 5, and 1 % levels, respectively

**Table 6:** Results from fixed and random effects estimation—higher recipient countries panel estimations—[Higher recipient countries]

Dependent variables	Fixed effects			Random effects		
	CRD	(D)BDP	(D)M2	CRD	(D)BDP	(D)M2
*Independent variables*						
(D) REM	−0.3017	0.1131	0.1532**	0.1015	0.1122*	0.1429*
	[−1.05]	[1.07]	[2.00]	[0.40]	[1.20]	[0.74]
(D) LOGGDP	−15.3153	−11.9440***	−5.1925*	−11.4134	−7.7320**	−9.626**
	[−4.32]	[‘−3.57]	[−1.66]	[−1.10]	[−2.03]	[−2.43]
(D) PCGDP	0.0678***	0.0015**	−0.0017*	0.0039*	0.0018	0.0004
	[17.63]	[2.05]	[‘−1.66]	[0.24]	[0.14]	[1.98]
INF	−0.0383	−0.0746***	−0.0369***	−0.0426*	−0.0656***	−0.0787***
	[−1.17]	[−6.11]	[−4.21]	[−1.30]	[−5.34]	[−7.07]
FDI	0.2153	0.0257	0.0308	−0.2771**	0.0051	0.0254
	[1.63]	[0.85]	[0.87]	[−2.05]	[0.01]	[0.57]
EXPORTS	0.0636	0.01855	0.0081	0.0311	−0.0047	0.0057
	[1.59]	[0.73]	[0.76]	[0.41]	[‘−0.08]	[0.42]
Constant	9.8354***	0.97447	0.8845	30.2127***	1.4434**	1.4571***
	[6.81]	[1.22]	[2.29]	[6.87]	[2.40]	[2.97]
R-squared	0.2947	0.3618	0.4035	0.3513	0.5204	0.5025
Number of observations	444	424	444	444	424	444
Number of countries	24	24	24	48	24	48
F-statistic	67.95	13.67	6.56			
Prob (F-statistic)	0.0001	0.0000	0.0000			
Wald chi 2				7.33	38.49	67.81
Prob > chi 2				0.0001	0.0000	0.0000

Absolute values of t-statistics are in brackets []. The symbol *, **, and *** denote significance at the 10, 5, and 1 % levels, respectively

In some and most instances, remittances are statistically significant as a positive determinant of bank deposits and money supply. The relationship between the availability of credit to private sector from the estimations were significant at 10 % levels and negative. The relationship between levels of remittances and bank deposits and money supply were positive and significant. For all the estimations in Africa, the size of the economy seems to be negatively related to financial development in all proxies. At significant levels, the size of the economy, proxied by the log of GDP is seen to be negatively related to the level of financial sector development measured by the credit to private sector as a percentage of GDP, percentage of bank deposits to GDP and quasi money to GDP. Similarly, per capita GDP seems to significantly affect financial development, though the magnitude of the effect is surprisingly small. Capital and current account openness are both associated with a greater financial development. But then, in some instances there were exhibits of a negative relation of capital and current account openness and financial development.

The coefficient of per-capita GDP is positive and significant at 1 and 5 % significant levels, consistently with the fact that economic development facilitates financial development. This in line with previous literature shows that economies with higher institutional and legal quality promoted the development of the financial sector.

The study included the rate of inflation, a measure of capital account liberalization or financial openness and a country‘s openness to international trade measured by the level of foreign direct investment flows into the region. The level of openness to international trade is measured by exports as a percent of GDP respectively. Intuitively, inflation discourages financial intermediation and therefore should result in lower financial development (Boyd et al. 2001). The results from all the estimations indicated a negative effect of inflation on financial development in all proxies. Inflation reduces the real value of assets and investments. Foreign direct investment from all the estimations indicated a positive effect on all the three proxies of financial development though they were rarely significant. Exports were positively related to financial development with the fixed and random effect estimations though not significant in some cases with the achievement of their expected signs. The link between financial development and remittances can be deemed as positive in all estimations. However, these estimates can be biased by endogeneity between financial development and remittances as earlier stated. To test the hypothesis of reverse causality and to deal with the issue of endogeneity, the study uses the VAR on the panel data.

To undertake this VAR, a cointegration test was performed to assess the existence or otherwise of a long term relationship between the three proxies of financial development and remittances. From the unit root tests in tables in the ‘Appendix’, the credit to private sector as a percentage of GDP as a proxy to financial development is stationary at levels i.e. I (0) whereas the other proxies bank deposit and money supply all, separately as percentage of GDP, and remittances are stationary at first difference. We use the Johansen cointegration technique to estimate the long run relationship for all the proxies of financial development but credit to private sector and remittances since these variables are all stationary at first difference. We adopt the use of the Autoregressive Distributive Lag (ARDL) approach proposed by Pesaran et al. (1998), Pesaran and Shin (1998). The results of these tests are represented below in Table 7, for credit to private sector as a percentage of GDP, and Table 8, for the other proxies of financial development.

**Table 7:** Wald test: cointegration test with ARDL

Test statistic	Value	Df	Probability
F-statistic	1546.342	(8, 657)	0.0000
Chi square	12,370.73	8	0.0000

Null Hypothesis: C(1) = C(2) = C(3) = C(4) = C(5) = C(6) = C(7) = C(8) = 0

**Table 8:** Johansen fisher panel Co integration tests

No. Of CE(s)		Fishers statistics				
Series		Trace test	Prob	Maximum Eigen value	Prob	No. of obs
BDP: REM	None	160.9	0.0000	142.9	0.0000	1100
	At most 1	112.1	0.0028	112.1	0.0728	
M2: REM	None	164.7	0	150.9	0.0000	1100
	At most 1	112.7	0.0025	112.7	0.0225	

*H*_*0*_ No cointegration exists

The results from Tables 9, 10 and 11 indicate that the coefficients of remittances are negatively related to financial development in the long run. The p values of the coefficient of the error correction equation are significant across all measures of financial development. This means remittances have a negative and significant long run relationship with the proxies of financial development.

**Table 9:** Credit to private sector as a percentage of GDP (CRD) and Remittances (REM)

Variables	D(CRD)	D(REM)
ECM(−1)	−0.0482***	−0.0029
	[−4.5344]	[−0.7196]
D(CRD(−1))	−0.0125	−0.0002
	[−0.3540]	[−0.0018]
D(CRD(−2))	0.0871***	0.0102
	[2.4060]	[0.7315]
D(CRD(−3))	0.0811***	−0.0009
	[2.3932]	[−0.0707]
D(CRD(−4))	−0.0128	0.0045
	[−0.3950]	[0.3610]
D(REM(−1))	−0.0374	−0.0553
	[−0.3700]	[−1.4094]
D(REM(−2))	0.0507*	0.0868**
	[0.5019]	[2.2129]
D(REM(−3))	−0.1155	−0.0876**
	[−1.1458]	[−2.2414]
D(REM(−4))	−0.0711	0.1278
	[−0.6994]	[3.2397]
DLOGGDP	6.7497***	−5.0112
	[3.4744]	[−6.6525]
DPCGDP	0.0043***	0.0001
	[6.5105]	[0.3918]
INF	−0.1806***	0.0005
	[−6.5086]	[0.0469]
FDI	0.0918	0.0645*
	[1.34670]	[2.4407]
EXP	−0.02397	−0.0079
	[−1.0978]	[−0.9336]
C	2.0198*	0.1848
	[2.4196]	[0.5711]
R-squared	0.3820	0.1134
Adj. R-squared	0.3636	0.0934
F-statistic	9.8879	5.6853
Akaike AIC	7.2879	5.3931

Figures in parentheses represent the t statistics.*, ** and *** denote 10, 5 and 1 % significance levels respectively

**Table 10:** Bank deposit as a percentage of GDP (BDP) and Remittances (REM)

Variables	D(BDP)	D(REM)
ECM(−1)	−0.0003**	0.0063***
	[−0.1970]	[5.1773]
D(BDP(−1))	0.0220*	0.0071
	[0.6455]	[0.2946]
D(BDP(−2))	−0.0271	0.0073
	[−0.7929]	[0.3013]
D(BDP(−3))	−0.0284	−0.0160
	[−0.8563]	[−0.6820]
D(BDP(−4))	−0.0698*	0.0082*
	[−2.1185]	[0.3517]
D(REM(−1))	0.0035**	0.0268
	[0.0618]	[0.6541]
D(REM(−2))	−0.0163	−0.0004
	[−0.2833]	[−0.0092]
D(REM(−3))	0.0876*	−0.0248
	[1.5085]	[−0.6031]
D(REM(−4))	0.0280	−0.0732*
	[0.4903]	[−1.8035]
DLOGGDP	2.9074**	−7.1305**
	[2.3553]	[−8.1326]
DPCGDP	0.0052**	0.0007
	[14.9815]	[0.2930]
INF	−0.1189**	0.0113**
	[−7.0888]	[0.9541]
FDI	0.0728	0.1308***
	[1.9207]	[4.8545]
EXP	−0.0102	−0.0101
	[−0.8287]	[−1.1547]
C	0.9604***	0.0174
	[2.0957]	[0.0535]
R-squared	0.3708	0.1995
Adj. R-squared	0.3550	0.1794
F-statistic	23.4953	9.9341
Akaike AIC	5.9610	5.2768

Figures in parentheses represent the t statistics.*, ** and *** denote 10, 5 and 1 % significance levels respectively

**Table 11:** Money supply as a percentage of GDP (M2) and Remittances (REM)

Variables	D(M2)	D(REM)
ECM(−1)	−0.0337***	0.0145***
	[−4.1134]	[2.9947]
D(M2(−1))	−0.0858**	0.0114
	[−2.5515]	[0.5735]
D(M2(−2))	−0.0275	0.0155
	[−0.8252]	[0.7831]
D(M2(−3))	−0.0469	0.0089
	[−1.4092]	[0.4533]
D(M2(−4))	−0.0508*	0.0075
	[−1.4333]	[0.3588]
D(REM(−1))	0.0590	−0.0371
	[0.8906]	[−0.9450]
D(REM(−2))	0.0249*	−0.0697**
	[0.3758]	[−1.7806]
D(REM(−3))	0.1214**	−0.0726*
	[1.8440]	[−1.8648]
D(REM(−4))	0.0373	−0.1129
	[0.5628]	[−2.8773]
DLOGGDP	5.6619***	−4.8123***
	[4.6214]	[−6.6394]
DPCGDP	0.0049***	0.0006
	[11.3663]	[0.2607]
INF	−0.1397***	−0.0024
	[−7.7608]	[−0.2203]
FDI	0.1119**	0.0739**
	[2.5564]	[2.8554]
EXP	−0.0002	−0.0061
	[−0.0173]	[−0.7329]
C	0.8562*	0.1052
	[1.5870]	[0.3297]
R-squared	0.3124	0.1213
Adj. R-squared	0.2971	0.1018
F-statistic	20.5712	6.2488
Akaike AIC	6.4159	5.3662

Figures in parentheses represent the t statistics.*, ** and *** denote 10, 5 and 1 % significance levels respectively

The study also assessed the short run causal relationship between remittances and financial development by using the Chi square value from the Wald test statistics. We assess this by evaluating the coefficients of the lags of remittances, as to whether at least one of the lags is not equal to zero. The short-run causality relationships can be tested through the coefficients of each lags of the explanatory variable which in our case, is either remittances or the proxies of financial development. The results are displayed in Tables 12 and 13.

**Table 12:** Short run causality from REM to FD: Wald Test

Dependent variables	CRD	BDP	M2
*Inde: REM(lags)*			
F-statistic	0.4228	0.6475	0.8419
Prob	0.7922	0.6288	0.4988
Chi square	1.6912	2.5899	3.3676
Prob > chi 2	0.7923	0.6286	0.4983

**Table 13:** Short run causality from FD to REM: Wald Test

Dependent variable	Remittances		
	CRD (1–4 lags)	BDP (1–4 lags)	M2 (1–4 lags)
F-statistic	0.1774	0.1604	0.2746
Prob	0.9500	0.9583	0.8944
chi 2	0.7098	0.6415	1.0983
Prob > chi 2	0.9501	0.9583	0.8945

All the proxies for financial development establish the fact that there is evidence of a short-run relationship from remittances to financial sector development, employing all proxies. The p values of the test tend not to reject the null hypothesis which states that at least one of the lags of remittances and financial development is not equal to zero and there is the existence of a short run causality of the lags of remittances on financial development. Again, on the other hand, the results from Table 13 use the Wald test to test for the short run causality that flows from the various proxies of financial development to remittances. The results reported indicate that at least one of the lags of the proxies of financial sector development does have a causal effect on the levels of remittances received by recipients in African countries in the short-run.

## Discussions

The African continent, evidently is known to be relegated in comparison to other developing areas. Countries in the Africa are mostly characterized by poor institutional qualities and the prevalence of poverty marked characteristics etc. The development of African countries hinges on the ability for these economies to convert every resource, revenue or grant for economic development. The study of the financial development of these countries, which is known to contribute immensely to economic growth is important. With increased number of migrants in recent years from Africa, it is deemed reasonable to see increased levels of remittances. With dire economic conditions compared to other developing countries, recipients rely greatly on these migrant funds for numerous reasons. Among the already mentioned reasons, how these funds can help promote the accessibility to credit facilities, indulgence in formal banking by recipients and the circulation of funds to promote the financial infrastructure, is what this study ventured to explore.

From the results gathered, the fixed and random effects estimations, used for all countries and higher recipient countries as baseline estimations indicated a negative and statistically significant at 10 % relationship between remittances and credit to private sector. This did not follow our hypothesis that remittances improved the availability of credit to the private sector. However, the results indicated a positive and significant relationship between remittances and bank deposits as well as money supply in the baseline estimations.

It is clearly noted that migrant remittances or transfers help ease the immediate budget constraints of recipient, and provide an opportunity for small savers to gain access to the formal financial sector. Remittances received can enable recipients who are unbanked to acquire certain financial products and services which will in turn improve financial sector development in the short run. From the study, these services from the financial institutions certainly are not skewed towards the availability of credit. Martinez et al. (2015) alluded to this fact, in their study of venture funding with remittances, that remittances did not specifically improve financial depth such as bank loan availability. Evidently in the context of Africa, receipts of remittances could not serve as a collateral or a guarantee to acquire a bank loan by individual recipients.

Remittances, from the empirical analysis, increased the saving pattern of recipients. Excess funds after consumption and use of other investments could be saved, introducing non banked recipients to formal banking and investment systems. This is consistent with other literature to an extent, (Gupta et al. 2009; Aggarwal et al. 2010). In the long-run however, recipients are able to side-step financial limitations that are imposed on or come with credit acquisition from financial institutions since recipients can wait to receive funds from ‘migrant-relatives’. This relationship of adverse impact of remittances on financial development in the long run could stem from the fact that remitted funds are not primarily for financial investment purposes or savings but are sent specifically for consumption purposes, which will not remain in the financial institution for a longer time, even if retained.

From the results from the error correction model estimates, there was a reverse causality from the measures of financial development to remittances. Despite the presence of some negative coefficients with the lags of the measures of financial development, there were others which were positive and significant. This is in line with the reverse causality assumption by previous literature and our hypothesis that better financial sectors in countries attracted remittances via the formal channels which are captured in appropriate records. Increased competition in the financial sector, as a result of financial sector development, according to previous literature, results in lowered transaction cost to remit money via formal channels may be a major reason for this reverse causality.

The use of the quasi money as a measure of financial sector development did prove to be somewhat but not much different from the other proxies with the recorded levels of significance. This was consistent with all the estimations used though some of the co-efficients are insignificant and negative with the second lag of remittances. The implication that can be derived out of this is that, remittances augment flow of money in circulation far more than ‘loanable’ funds or funds primarily intended for deposit or the purchase of any financial product in Africa. Again, the notion that remittances received may serve as a platform to acquire financial assistance in the form of loans is not supported by the findings of this study. The just mentioned reasons are deemed very viable because remittances are more likely to be used for consumption and other purposes than being saved in the long-run by reason of deferred consumption.

## Conclusion

 This essay attempted to empirically establish the effect of remittances on financial development in Africa. The study finds that remittances have a positive impact on financial development in the short-run but a negative effect in the long run with credit to private sector as a measure of financial development, bank deposit and mostly money supply.

The findings generally indicated that remittances positively and significantly influence certain aspects of financial development such as bank deposits and money supply, leaving out the eligibility to acquire credit from banks in Africa. However, these remittances received however did not promote financial development in the long-run. This scenario is real since remittances are basically used for survival purposes by the recipient. In line with some previous studies, remittances could be primarily purposed for meeting basic needs such as education, clothing, housing and entrepreneurial ventures etc. by the recipients or the migrants’ home countries. These uses of remittances are not promoters of immediate or contemporaneous development of financial development, particularly using credit to private sector and bank deposits, all percentages of GDP and as proxies of financial development. This is due to the fact that remittances received are not long left with financial institutions but for other purposes.

The study reveals that financial development caused more remittances for the period under review. Hence, looking at the role of remittances in Africa, as discussed at the outset of the study, the development of the financial sector can help increase the propensity to remit.

The increased adoption of money transfer operations by some financial institutions is an indication that when and if remittances are sent through formal channels certain aspects of the financial sector could improve over time. Financial institutions can adopt money transfer operators and various fund transfer mechanisms in order to introduce unbanked recipients to some financial products and services. This could go a long way to improve the financial sector and promote financial inclusion in most developing countries, particularly in Africa.

### Implications and limitations of study

Our findings add to the pool of studies concerning remittances and contribute to ideas concerning credit availability, bank deposit and inclusions and money supply promoted by migrants’ remittances. There is less evidence to concretize the assumption that increased remittances may guarantee accessibility to credit in Africa. The exposure to banking systems and products in the short run may be a viable outlook. Practitioners and policy makers may draw from this research, a way to link remittances received via formal ways to the advancement of banking products and services. The intuition of increased remittances via the formal sector by reducing transaction cost may be deemed an option to pursue. Credit facility programs could be adopted by financial institutions, which have money transfer operations as a business along their core modus operandi. Policy makers in governments and other international institutions may formulate policies concerning the financial infrastructure of countries can be tailored to include recipients of remittances who may simply rely on these funds for consumption. Wealth creation programmes could be advanced to them and include unbanked recipients into the financial bracket.

Our limitations to this study may suggest areas which future research may have a look at. The study did not account for intra-regional remittances which could also form a major part of remittances received by African countries. The study did not use the concept of different exchange rate regimes as a control variable for which further studies may inculcate. Finally, measures of financial development may not fully encompass all aspects of the financial infrastructure in African countries as there is a surge of financial markets and other measures of financial development.

## Appendix

See Tables 14, 15 and 16.

**Tables 14:** Units root test of panel data

Variable	ADF	PP
*(a) Unit root tests at levels*		
CRD	188.046 [0.0000***]	147.063 [0.0001***]
BDP	74.7735[0.9611]	108.266 [0.2247]
M2	102.197[0.8432]	79.8639[0.8760]
REM	110.473 [0.1519]	115.876 [0.0818*]
PCGDP	24.8632 [1.0000]	16.1173 [1.0000]
LOGGDP	12.3640 [1.0000]	17.723 [1.0000]
INF	382.192 [0.0000***]	421.447 [0.0000***]
FDI	257.796 [0.0000***]	241.607 [0.0000***]
EXP	138.533 [0.0044***]	126.6071 [0.0275**]
*(b)Unit root tests at First Differencing*		
BDP	610.372 [0.0000***]	731.285 [0.0000***]
M2	514.287 [0.0000***]	647.593 [0.0000***]
REM	469.343 [0.0000***]	708.781 [0.0000***]
PCGDP	332.315 [0.0000***]	431.134 [0.0000***]
LOGGDP	435.274 [0.0000***]	476.631 [0.0000***]

p values are in brackets []. The symbol *, **, and *** denote significance at the 10, 5, and 1 % levels, respectively

**Table 15:** Test for autoregressive conditional heteroskedasticity

Dependent variables	CRD	BDP	M2
F-statistic	0.431475	0.003677	0.894654
Prob	0.6498	0.9517	0.3446
Obs*R-squared	0.866049	0.00369	0.896201
Prob > chi 2	0.6485	0.9516	0.3438

**Table 16:** Test for serial correlation in residuals

Dependent variables	CRD	BDP	M2
F-statistic	0.879672	13.24484	23.62508
Prob	0.4154	0.2212	0.3124
Obs*R-squared	1.801321	26.07106	45.20021
Prob > chi 2	0.4063	0.2109	0.3012
